# Monitoring the long term vegetation phenology change in Northeast China from 1982 to 2015

**DOI:** 10.1038/s41598-017-14918-4

**Published:** 2017-11-07

**Authors:** Lingxue Yu, Tingxiang Liu, Kun Bu, Fengqin Yan, Jiuchun Yang, Liping Chang, Shuwen Zhang

**Affiliations:** 1 Northeast Institute of Geography and Agroecology, Chinese Academy of Sciences, Changchun, 130012 P. R. China; 2 Changchun Normal University, Changchun, 130031 P. R. China

## Abstract

Global warming has contributed to the extension of the growing season in North Hemisphere. In this paper, we investigated the spatial characteristics of the date of the start of the season (SOS), the date of the end of the season (EOS) and the length of the season (LOS) and their change trends from 1982 to 2015 in Northeast China. Our results showed that there was a significant advance of SOS and a significant delay of EOS, especially in the north part of Northeast China. For the average change slope of EOS in the study area, the delay trend was 0.25 d/y, which was more obvious than the advance trend of −0.13 d/y from the SOS. In particular, the LOS of deciduous needleleaf forest (DNF) and grassland increased with a trend of 0.63 d/y and 0.66 d/y from 1982 to 2015, indicating the growth season increased 21.42 and 22.44 days in a 34-year period, respectively. However, few negative signals were detected nearby Hulun Lake, suggesting that the continuous climate warming in the future may bring no longer growing periods for the grass in the semiarid areas as the drought caused by climate warming may limit the vegetation growth.

## Introduction

Vegetation phenology is an important indicator for monitoring changes in the climate and natural environment^1–3^. Studies of vegetation phenology are of great significance to understand the change trend of natural seasonal phenomena and serve for agricultural production and global change studies^4–6^. Vegetation phenology is very sensitive to climate fluctuation^1,2^. Climate change directly affects the growth and development of plants as well as the geographical distribution patterns of plants^7–9^. In addition, vegetation phenology is also an important parameter for land surface process models and the global carbon cycle^10^. Accurate monitoring of the vegetation phenology can help explore the evidence for a response of the vegetation to climate change, enhance the understanding of the material and energy exchange between the vegetation and climate and accurately evaluate the vegetation productivity and global carbon budget^11,12^.

At present, the phenological observation methods mainly include ground observation and remote sensing monitoring^13,14^. Though traditional ground observation is objective and accurate, it cannot generate large-scale continuous surface observation data^15^. The satellite multispectral sensor can perform repeated observations in a short time period, which provides a data source for vegetation phenology research at both the community and regional scale^16^. The normalized difference vegetation index (NDVI) derived from remote sensing data can reflect the greenness, metabolic intensity and seasonal and interannual variation of vegetation, and it has been widely used for indicating vegetation characteristics such as vegetation coverage, vegetation type, and leaf area index as well as for monitoring the seasonal variation of vegetation and land cover changes^17,18^. The methods used to detect the start and end of the growing season based on time series NDVI data mainly contains the following categories: dynamic or static threshold method^19,20^, maximum slope method^21^, curve fitting method^22,23^ and empirical regression equation method^24^. Among these methods, some are difficult to apply on the regional scale and are limited by the local experience parameters of the study area, while others lack of enough analysis of the phenological patterns for specific ecosystems but can obtain regional and even global-scale phenological patterns^25^.

The temperature records of the 20th and 21st centuries show that global temperatures have clearly increased since the 1970s, especially in the middle and high latitudes areas of the Northern Hemisphere^26^. The increase of temperature has led to the advanced spring phenology, postponed autumn phenology, and extension of the growing season in this region^27–29^. Northeast China, and more generally, the northern part of China, is extremely sensitive to the global climate change^4,30^. Previous studies have shown that the phenology of typical vegetation such as forests and crops in Northeast China has changed. These studies only illustrated the phenological variation characteristic for each typical type but lack a spatial comparative analysis across the study area, which is essential as northeast China covers cold, middle and warm temperate zones and heterogeneous vegetation types. It is of great significance to describe the changes in the vegetation phenology in a continuous regional space for studying the response of vegetation phenology to global climate change.

In this paper, we first extracted the vegetation phenological variables using a double logistic model based on the GIMMS NDVI dataset and the phenological field observation data, and then we used the linear regression and least square method to extract the change trend for the phonological parameters such as SOS, EOS and LOS. Finally, on the basis of land cover data, we calculated and compared the vegetation phenology change between different vegetation types through statistical analysis. Our study not only illustrated the spatial variation trend of the vegetation phenology but also clarified the vegetation phenological characteristics for different vegetation types in Northeast China. This study can provide definite evidence for global climate change and natural environment change.

## Results

### Validation of the extracted vegetation phenology parameters

Based on the long-term phenological observation records of 8 stations from 1982 to 2008, we compared the SOS and EOS of the observation values with the simulated results. A function *y* = *x* was used to evaluate the accuracy of the results and the bias error^31^. To make the results more comparable, we selected nearly pure pixels for different vegetation types around the stations to calculate and evaluate.

The root mean square error of the simulated SOS and EOS were 3.14 days and 3.90 days. As the resolution in our study was 8 km, the scale mismatch between 8 km resolution and point may be the main error source. As most of the stations located in the center of the city, it was difficult to find the pure pixels there because of the significant heterogeneity of land cover at 8 km resolution. As a result, we selected the nearest pure pixels around the stations but may bias from the center of the city where under the influence of an urban heat island effect, i.e., a cooling effect, may be observed from the pure pixels and their nearest stations^32,33^. In our results, the extracted SOS were slightly higher, and the extracted EOS were slightly lower than were those from stations, suggesting that the extracted growing season was shorter than that from the stations, which was consistent with the above analysis.

Nonetheless, both the point pairs in the SOS and EOS are similar to the function *y* = *x*, which indicates good consistency between the simulated and observed data. Meanwhile, we analyzed the bias error in different years and found that there was no trend for the root mean square error from 1982 to 2008. Therefore, we assume that our vegetation phenology extraction model and threshold can obtain relatively accurate SOS and EOS values in Northeast China.

### Vegetation phenology characteristics in Northeast China

**Figure 1:**
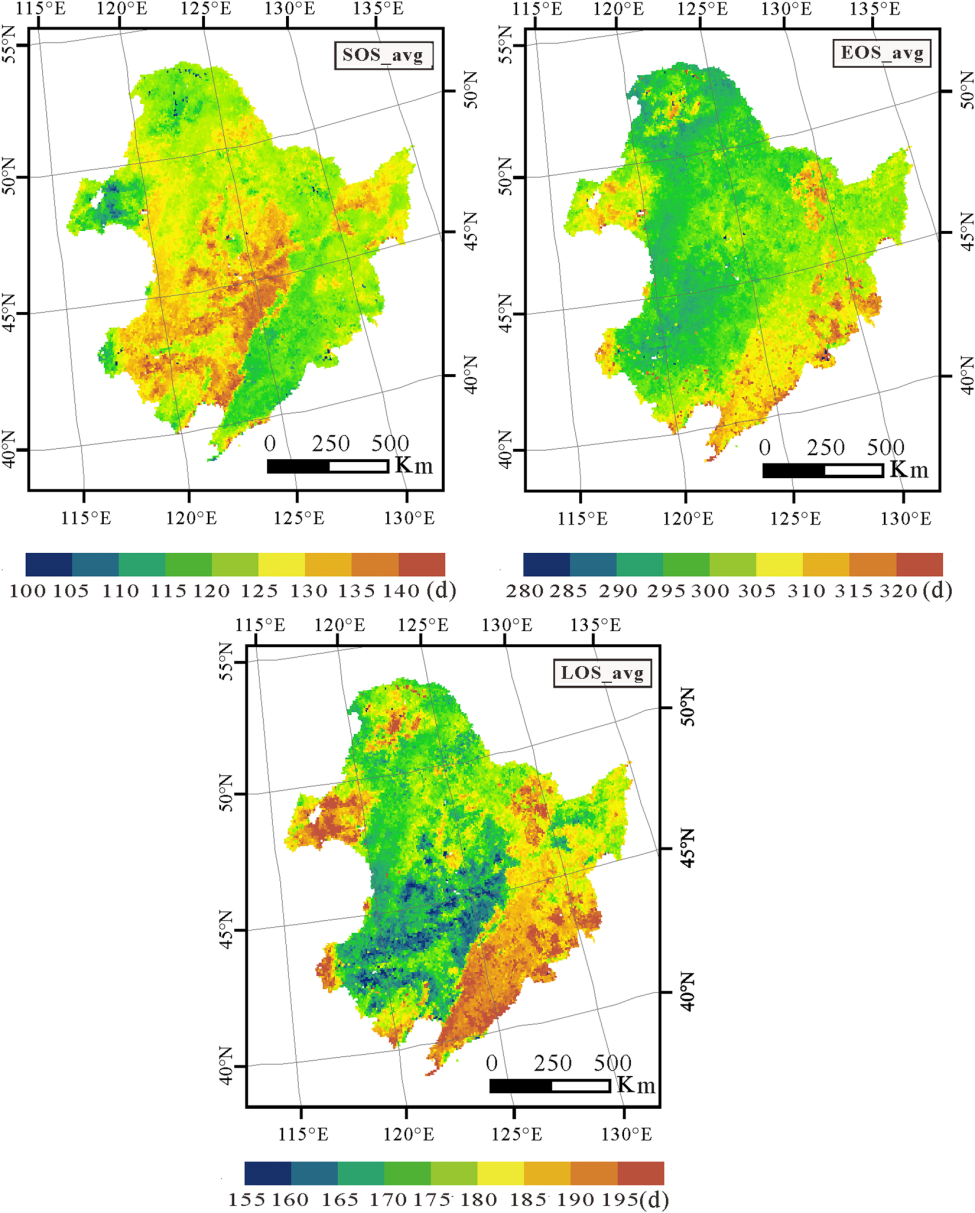
The spatial distribution of the averaged SOS (SOS_avg), averaged EOS(EOS_avg) and averaged LOS(LOS_avg) from 1982 to 2015. (This figure was drawn by ArcGIS10 http://www.esri.com/arcgis/).

Based on the vegetation phenology extraction model, the spatial distribution of the SOS, EOS and LOS were obtained from 1982 to 2015 in northeast China. To eliminate the effect of mutation data years on the results, we averaged the SOS, EOS and LOS from 1982 to 2015 to express the characteristics for the vegetation phenology in Northeast China (Fig. 1).

The spatial distribution characteristics for the vegetation phenology are illustrated in Fig. 1. All three phenology parameters (SOS, EOS and LOS) showed significant spatial heterogeneity as well as differences between each other. In general, the SOS in Northeast China from 1982 to 2015 ranged from the 100^th^ day to the 140^th^ day of the year, respectively, representing April 10^th^ and May 20th. Similarly, the EOS in Northeast China from 1982 to 2015 ranged from the 280^th^ day to the 320^th^ day in the year, respectively representing October 7^th^ and November 16th. As a result, the average LOS ranged from 115 days to 195 days. In addition, the results suggested that there existed some relationship between phonological parameters and land cover types.

### Vegetation phenology changes in Northeast China

**Figure 2:**
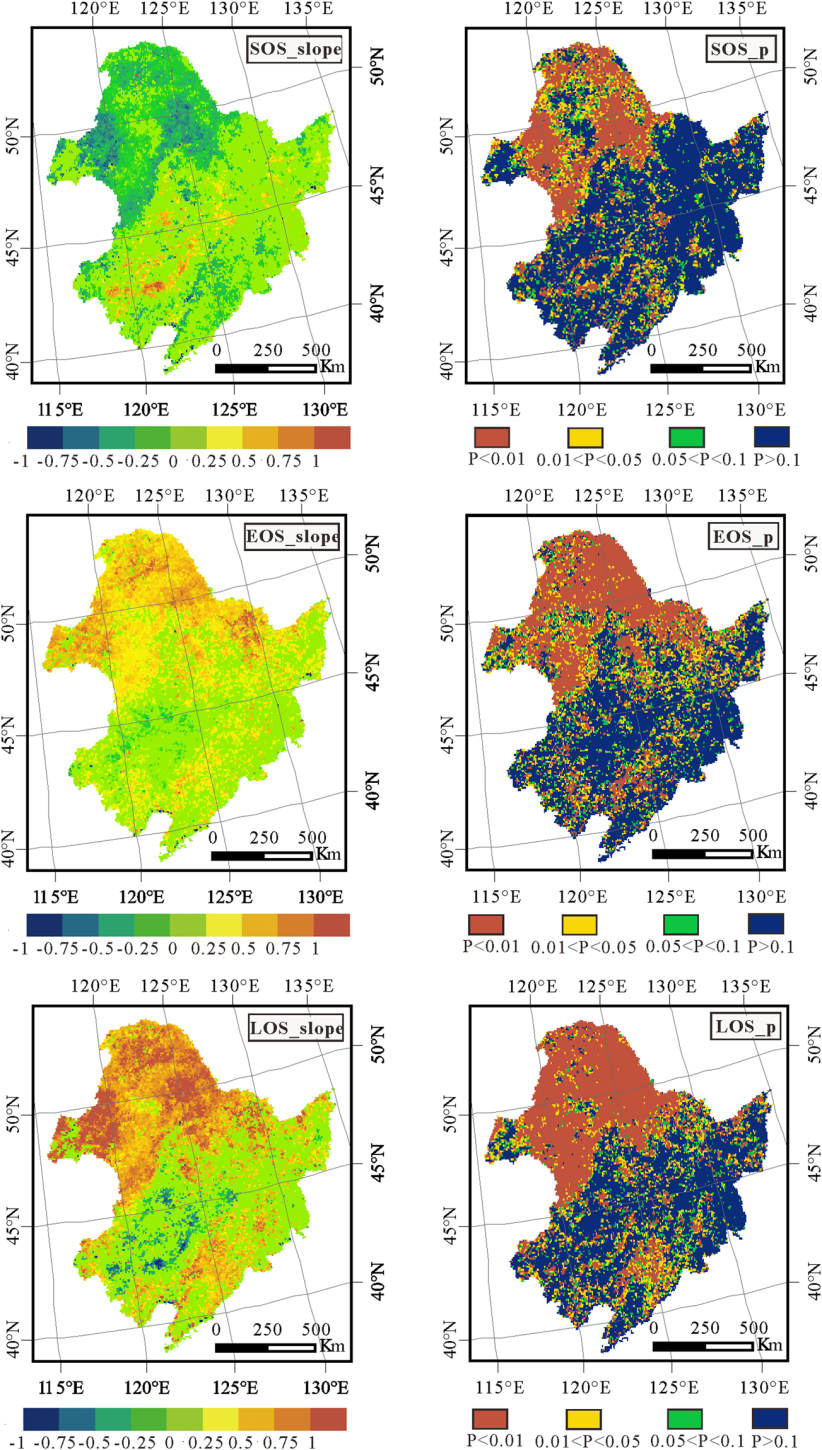
The slope of the SOS(SOS_slope), slope of the EOS(EOS_slope), and slope of the LOS(LOS_slope) and the corresponding confidences of the change trend for SOS, EOS and LOS (SOS_p, EOS_p, LOS_p, respectively) from 1982 to 2015 in northeast China (a trend of zero lies outside the 90% confidence interval). (This figure was drawn by ArcGIS10 http://www.esri.com/arcgis/).

The change trends for the SOS, EOS and LOS and the corresponding confidences from 1982 to 2015 were extracted based on the multi-SOS, multi-EOS and multi-LOS layers (Fig. 2). In the results, only the change trends lying inside the 90% confidence interval were shown in the slope maps, and the trends without significance were set to zero in the slope maps.

The SOS_slope and SOS_p shown in Fig. 2 illustrated that there are significant advances in the growth season, especially in the north part of Northeast China. Forests on both sides of Greater Khingan Mountain, the grassland in Hulun Buir and the farmland at the northern Nenjiang plain showed a −0.25 d/y or more negative trend. The natural climate change has been proven to be the dominant factor for the advance in SOS^27,28^. The north part of Northeast China was the most significant region for climate warming^4^, which contributed to the significant earlier trend in the SOS. The EOS_slope and EOS_p showed a postponement trend of the growing season in most parts of Northeast China, especially for the northernmost part, which was similar to the SOS but with a larger extent. The climate warming in autumn may lead to the later EOS for larger areas as well as more types. The changes in SOS and EOS contributed to the changes in LOS. The LOS in Northeast China showed an obvious increase in most parts of the study area. In the northern part of the study area, the LOS increased with a trend larger than 1 d/y, i.e., the growth season increased by more than 30 days during 1982 to 2015. As a result, the farmland at the northern Nenjiang plain converted its crops from soybean to corn in 2010 ^34^, which can provide evidence for the increase of the LOS in the northern Songnen Plain.

In addition, there are areas with a shorter LOS that are centrally distributed at Songnen and Liaohe Plain. The overlay analysis between LOS_slope and land cover maps indicated that the LOS with significant decreasing trends were mainly located at the transition zone between farmland and grassland. The conversion from grassland to farmland due to human activities in these areas during 1982 to 2015 may be the main reason for these results. The land use change study in northeast China from Zhang *et al*. (2006) and Wang *et al*. (2009) indicated that the conversion dominated the change^35,36^.

Based on the land cover maps, we extracted the change characteristics for the six main types to clarify the relationships between vegetation cover types and the change slope of the SOS, EOS and LOS at a confidence level. To eliminate the influence from mix pixels on the results, the near pure pixels with the area percentage higher than 80% were utilized in the statistics and analysis. The values lying outside the 90% confidence interval were set to zero in the statistics. The number of grids involved in the operation, the average slope and the percentages of the grids with significance at P < 0.1 for the SOS, EOS and LOS for each land cover type were then calculated and are shown in Table 1.

**Table 1:** The change trend of the SOS, EOS and LOS (*_slope) for six land cover types (Deciduous Needleleaf Forest (DNF), Deciduous Broadleaf Forest (DBF), Mixed Forest (MF), Grass, Farm and Swamp) and the corresponding grid proportions with significance at P < 0.1(*_per).

Type	Count	Sos_slope	Sos_per	Eos_slope	Eos_per	Los_slope	Los_per
DNF	1279	−0.24	78.58	0.36	92.26	0.63	95.00
DBF	1583	−0.09	35.38	0.17	53.19	0.28	57.74
MF	749	−0.04	19.63	0.35	55.01	0.31	45.39
Grass	975	−0.28	58.87	0.28	74.87	0.66	73.95
Farm	3464	−0.02	39.67	0.13	46.13	0.15	46.33
Swamp	37	−0.12	29.73	0.23	59.46	0.31	51.35

The near pure pixels for each land cover were analyzed in this section, including 1279 grids for DNF, 1583 grids for DBF, 749 grids for MF, 975 grids for Grass, 3464 grids for Farmland and 37 grids for swamp. The number of the pixels can not only reflect the area and fragmentation degree for each land cover to a certain extent but can also show the rationality of the statistic as there is no statistical significance if the sample is too small^37^. In this study, the swamp pixels were relative small and were not considered as a primary object in the analysis.

The SOS for each land cover type showed an advanced trend. The change slope for grassland was lowest, with 0.28 days advanced per year and 58.87% significant pixels. The deciduous needleleaf forest changed at a rate of −0.24 d/y, in which 78.58% pixels were significant at P < 0.1. The other types showed a slower decrease trend as well as a higher percentage of pixels with no significance. In contrast to the SOS, the EOS indicated a delay trend and a higher change rate. The EOS for DNF was delayed at a rate of 0.36 d/y, in which 92.26% pixels were significant at a 90% confidence interval, i.e., the EOS of DNF in the study area extended approximately 12 days for the EOS from 1982 to 2015. The EOS for MF and Grassland also showed an obvious increasing trend, respectively 0.35 d/y and 0.28 d/y. The change rate for the EOS of farmland and the DBF was higher than that for the SOS. In addition, the grid proportions with significance for the EOS were higher than those of the SOS for each land cover type, suggesting a stronger change trend and higher significance for the EOS. As a result, the growing season became longer, with a higher change rate and significance. The LOS of DNF increased with a trend of 0.63 d/y from 1982 to 2015, indicating that the growth season increased 21.42 days in a 34-year period. Meanwhile, 95% of pixels were significant at the 95% confidence level. The average slope of the LOS for grassland was 0.66 d/y, approximately 22.44 days longer in 2015 than in 1982. The increase trends for DBF, MF and farmland were 0.28 d/y, 0.31 d/y and 0.15 d/y respectively, and more than 40% of pixels were significant at P < 0.1.

## Discussion

Many previous studies have reported an earlier SOS, delayed EOS and longer LOS in the Northern Hemisphere^38–40^. Our results showed similar trends at longer temporal scales from 1982 to 2015. In Europe, the trends for an earlier SOS were 0.56 days per decade, and the delayed EOS was 9.6 days per decade during the period from 1982–2001 that was investigated by Stockli and Vidale^39^. The study from Chen (2005) also showed an apparent extension of the growing season, with 1.4 days per year across temperate eastern China during 1982–1993 ^41^. Our studies showed less of an increasing trend for the growing season from 1982 to 2015, which suggested that the increase trend for the growing season was slowing at some times during our study periods. It is interesting that the study from Jeong showed that the SOS advanced by 5.2 days during 1982 to 1999 but advanced by only 0.2 days during 2000 to 2008 in the north hemisphere^40^, indicating a lower increasing trend since 2000. To clarify the difference in the change trajectory for the growing season during different periods, the temperature and precipitation trends should be incorporated into further studies.

The vegetation phenology was different for different ecosystems^25^. The previous change trend studies for the growing seasons focused mainly on the regional averaged trend^12,42^ while ignoring the inner spatial diversity between ecosystems. In our study, we analyzed and distinguished the change trend for each land cover type, which showed great diversity between different types. It should be noted that in the United States, the growing season for the forest whose growth is normally limited by low temperatures and short growing seasons appears to be increasing, while the semiarid forest seems to be decreasing due to the longer drought and warm periods^27,43,44^. In Northeast China, the change trend of the LOS for almost all the forests was positive, which may have contributed to the geographical conditions because the forests in this area grew up in relatively humid areas^31^. Even the change trend for grassland in the semiarid areas did not show obvious negative signals, indicating that the rising temperature was beneficial for the growth of most forages. However, few negative signals were detected nearby Hulun Lake, suggesting that the continuous climate warming in the future may bring no longer growing periods for the grass in the semiarid areas as the drought caused by climate warming may limit the vegetation growth.

From another prospective, compared with previous phenology studies in Northeast China^4,11,25^, this study calculated the change slope for each nearly pure pixel and estimated the change trend for each land cover type based on a spatial statistics analysis, which was more credible for illustrating the spatial variation characteristics of the vegetation phenology. The results showed that higher change slope and change confidence of LOS in the regions with less human disturbance, suggesting the climate change was the dominant contributor for the phenology changes, which was consistent with the studies from Tang^5^ and Chen^41^. However, according to the results in Table 1, the change slopes of many pixels were not significant. Is the vegetation itself not changed significantly, or was the difference caused by external factors? For example, this uncertainty may have contributed to the land cover changes from natural vegetation to cultivated vegetation during 1982 to 2015 as the human activity was obvious in this period^34,45^. This hypothesis can help explain why the pixel numbers with a significant change in the farmland were lower than for other types, but further study is required in the future.

## Materials and Methods

### Study area

**Figure 3:**
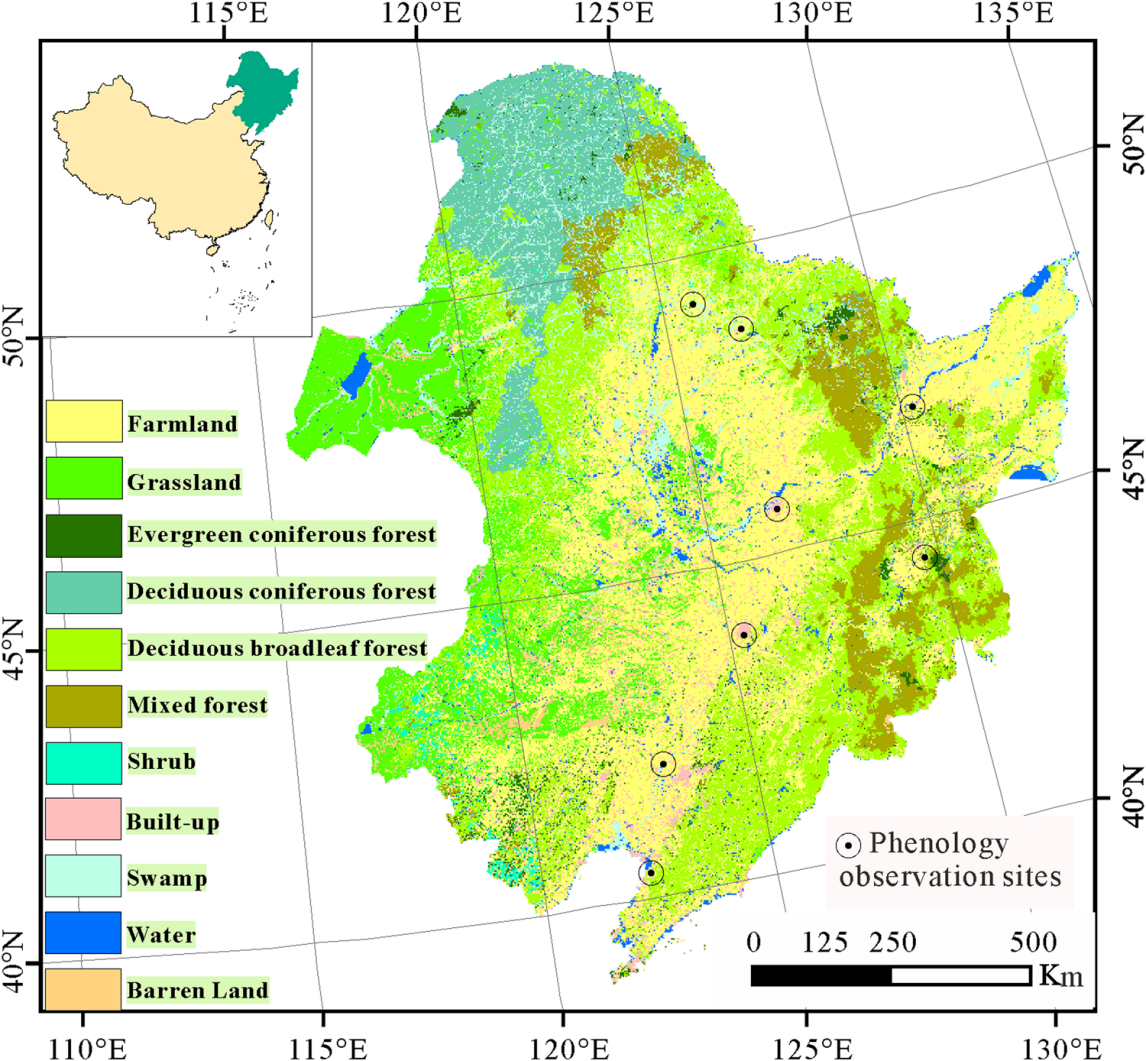
The land cover status of 2015 for 8 phenology observation sites in Northeast China (this figure was drawn by ArcGIS10 http://www.esri.com/arcgis/).

Northeast China is located in the eastern margin of the Eurasian continent, which ranges from 115°05′E to 135°02′E in longitude and from 38°40′N to 53°34′N in latitude, including Liaoning province, Jilin province, Heilongjiang province and 5 Cities of Inner Mongolia (eastern Mongolia). The study area is located in the temperate and monsoon climatic zone with a typical continental climate. The average annual precipitation and temperature are respectively approximately 400–700 mm and −1.1 °C–4.4 °C, with a long, extremely cold, and dry winter and a short, mild and moist summer. The coexistence of forest, pasture and agricultural land exhibits a mosaic and fragmental pattern of land use (Fig. 3), making the study area very sensitive to climate change.

### Methods for extracting the vegetation phenology

In this study, we used double logistic functions to fit the local seasonal variation of the vegetation. The local model functions can be expressed as follows:

1 $$f(t)={c}_{1}+{c}_{2}\,g(t;x)$$

where the linear parameters c_1_ and c_2_ determine the base level and the amplitude of the fitting model, respectively. The on-linear parameters *x*(${x}_{1},{x}_{2},{x}_{3},{x}_{4}$) determine the shape of the basis function *g(t*; ${x}_{1},{x}_{2},{x}_{3},{x}_{4}$). The basis function is a double logistic function^46^:

2 $$g(t;{x}_{1},{x}_{2},{x}_{3},{x}_{4})=\frac{1}{1+\exp (\frac{{x}_{1}-t}{{x}_{2}})}-\frac{1}{1+\exp (\frac{{x}_{3}-t}{{x}_{4}})}$$

where *x*_1_ and *x*_3_ determine the position of the left and right inflection point respectively, while *x*_2_ and *x*_4_ give the rate of change at the corresponding inflection points. The unknown parameters *c(c*_1_, *c*_2_) and *x(* ${x}_{1},{x}_{2},{x}_{3},{x}_{4}$) can be obtained through separable non-linear least-squares fits. Given a set of data points *(t*_*i*_, *y*_*i*_ *)*, *i* = *n1*,*…n2* that is located in an interval around the inflection points, the parameters *c* and *x* are obtained by minimizing the merit function:

3 $$M=\sum _{i=n1}^{n2}{[{w}_{i}\ast f({t}_{i})-{y}_{i}]}^{2}$$

where *w*_*i*_ is the weight of the data point. In this study, *w*_*i*_ is extracted from the percentile file containing the data quality information. We set the *w*_*i*_ to 1 when the data point is an acceptable value, 0.5 when the data point is an interpolation value and 0.1 when the data point is a seasonal extraction value.

The local model functions were merged at the full interval to generate the global function, which can realize the fitting of the long time series data. In this study, we used TIMESAT3.2 to conduct the time series fitting and the output of the phenological parameters. For the fitted time series, a dynamic threshold of 0.2 was used to extract the SOS, EOS, and LOS.

### Methods of analysis

For the long time series vegetation phenological parameters, we used the linear regression method to analyze the spatial variation characteristics. During the linear regression, the least square method was used to find the best match function for the data through minimizing the sum of the squared error. The slope of the fitting function can be expressed as follows:

4 $$S=\frac{n\times \sum _{i=1}^{n}i\times VP{P}_{i}-\sum _{i=1}^{n}i\,\sum _{i=1}^{n}VP{P}_{i}}{n\times \sum _{i=1}^{n}{i}^{2}-{(\sum _{i=1}^{n}i)}^{2}}$$

where ${{VPP}}_{i}$ is the vegetation phenological parameter value for year *i*, and *n* is the years during the monitoring period. *S* is the slope for the changing tendency during the study period. *S* greater than 0 indicates an increase trend, while less than 0 indicates a reduction trend. We conducted statistical tests based on variance analysis, and only the change trend is significant (with P < 0.1) and can be illustrated and analyzed. The data process was based on the R software. Figures 1, 2 and 3 were drawn by ArcGIS10 (http://www.esri.com/arcgis/).

### Data acquisition and availability

The GIMMS (Global Inventory Modeling and Mapping Studies) NDVI (normalized difference vegetation index) time series data were used to extract the vegetation phenology information. The GIMMS NDVI dataset is a global vegetation index dataset available for a 35-year period spanning from July in 1981 to December, 2015. The dataset was derived from Advanced Very High Resolution Radiometer (AVHRR) images that have been corrected for calibration, view geometry, volcanic aerosols, and other effects not related to vegetation change. The time resolution is 15 days, and the spatial resolution is 8 km. In this study, we used the GIMMS data (version 3 g.v1; https://ecocast.arc.nasa.gov/data/pub/gimms/3g.v1/) from 1982 to 2015. The 3 g.v1 data contains the NDVI as well as the percentile information. The actual NDVI value can be extracted by DN/10000. In the percentile layer, the flags equal to 0 represented the data points that were acceptable values, flags equal to 1 represented the data points that were obtained through a spline interpolation, and flags equal to 2 represent the data points that were obtained from seasonal profiles. The flag data were used to determine the fitting weight in the vegetation phenology extraction model. The projection was finally transferred to Albers from WGS84 after the extraction of vegetation phenology parameters.

The land cover data used in this study were obtained through human-computer interactive interpretation based on Landsat 8 images with an overall accuracy above 90%. The classification system for this dataset includes 7 first classes, including forest, grassland, farmland, built-up, wetland, water and other land, and 28 secondary classes. In northeast China, the land cover types cover all the first classes and 25 secondary classes. As the spatial resolution for this dataset was 30 meters and was 8 km for the GIMMS data, mixed pixels at 8 km resolution were inevitable. Therefore, we extracted the percentage for each land cover type at 8 km, and only the percentage greater than 80% can be used to analyze the phenological characteristics for different vegetation types. Then, only the main types can be used to analyze the relationship between vegetation types and phenological variables. To make the results statistically significant, we chose the land cover types with more than 100 high percentage (greater than 80%) pixels as the main types.

The phenological observation data are from the long-term observation records of 44 typical stations from the Chinese Phenological Observation Network (http://www.geodata.cn). The representative plants in each station were selected according to the principle of representative species, long observation time and good continuity to record the typical phenological period. The following 8 typical stations covering the study area from 1982 to 2008 were available for our study: Haerbin, Jiamusi, Mudanjiang, Nenjiang, Dedu, Changchun, Shenyang and Gaizhou. We set the beginning date of leaf expansion and leaf fall as the start and end times of the season, respectively. The phenological observation data were used to validate the accuracy of extracting phenological variables.
